# Evaluating Hole Quality in Drilling of Al 6061 Alloys

**DOI:** 10.3390/ma11122443

**Published:** 2018-12-02

**Authors:** Mohammad Uddin, Animesh Basak, Alokesh Pramanik, Sunpreet Singh, Grzegorz M. Krolczyk, Chander Prakash

**Affiliations:** 1 School of Engineering, University of South Australia, Mawson Lakes 5095, SA, Australia; Mohammad.Uddin@unisa.edu.au; 2 Future Industries Institute, University of South Australia, Mawson Lakes 5095, SA, Australia; 3 Adelaide Microscopy Unit, University of Adelaide, Adelaide 5005, SA, Australia; animesh.basak@adelaide.edu.au; 4 Department of Mechanical Engineering, Curtin University, Perth 6845, WA, Australia; alokesh.pramanik@curtin.edu.au; 5 School of Mechanical Engineering, Lovely Professional University, Phagwara, Punjab 144411, India; snprt.singh@gmail.com; 6 Department of Manufacturing Engineering and Automotive Products, Opole University of Technology, 76 Proszkowska St., 45-758 Opole, Poland; g.krolczyk@po.opole.pl

**Keywords:** drilling, dynamometer, hole quality, forces, roundness, roughness, wear, chips, burr

## Abstract

Hole quality in drilling is considered a precursor for reliable and secure component assembly, ensuring product integrity and functioning service life. This paper aims to evaluate the influence of the key process parameters on drilling performance. A series of drilling tests with new TiN-coated high speed steel (HSS) bits are performed, while thrust force and torque are measured with the aid of an in-house built force dynamometer. The effect of process mechanics on hole quality, e.g., dimensional accuracy, burr formation, surface finish, is evaluated in relation to drill-bit wear and chip formation mechanism. Experimental results indicate that the feedrate which dictates the uncut chip thickness and material removal rate is the most dominant factor, significantly impacting force and hole quality. For a given spindle speed range, maximum increase of axial force and torque is 44.94% and 47.65%, respectively, when feedrate increases from 0.04 mm/rev to 0.08 mm/rev. Stable, jerk-free cutting at feedrate of as low as 0.04 mm/rev is shown to result in hole dimensional error of less than 2%. A low feedrate along with high spindle speed may be preferred. The underlying tool wear mechanism and progression needs to be taken into account when drilling a large number of holes. The findings of the paper clearly signify the importance and choice of drilling parameters and provide guidelines for manufacturing industries to enhance a part’s dimensional integrity and productivity.

## 1. Introduction

In manufacturing industries, hole drilling has been a signature process employed to create various geometric features as well as to ensure secure assembly with other components for enhanced product integrity, reliability, and life cycle [1,2]. In particular, the process has been one of the major fabrication processes in automotive and aerospace industries, when machining of lightweight metals and composites are concerned [3,4]. Tool-based and laser-assisted drilling are adopted to fabricate the holes with the desired hole quality [5]. While laser drilling is shown to create holes with high geometric precision, often high process temperature may potentially deteriorate the structural integrity of the part. Such a phenomenon has unanimously been touted as a major issue in drilling of the composites. For instance, high temperature causes melting and swelling around the hole area, thus leading to damage to the drilled part [6].

In particular, burr formation and poor surface quality in drilling negatively affect the dimensional accuracy, and cause additional difficulty, reworking, cost, and even damage, e.g., fatigue, in the assembly. Therefore, the drilled holes are often deburred to retain a component’s functional reliability. It is reported that the deburring simply accounts for about 30% of total fabrication cost in an aircraft’s fuselage assembly [7]. As such, the importance of minimization of burr formation and comprehensive techniques to achieve this have been stressed out, with an aim of developing a more robust process modelling and database.

Regardless of materials and techniques, the important process parameters, such as spindle speed and feedrate, significantly affect the drilling performance, in terms of material removal rate, thrust force, and torque. The effect of these parameters on the process mechanics and their optimization in the drilling of different types of materials has been studied [8]. It is shown that the thrust force and torque dictates the final outcome of the drilling. A singled-out consensus, though, is that high thrust force and torque result in poor hole quality and deterioration of tool life. It is, therefore, very important to assess and understand further the process mechanics in a drilling process.

Commercial piezoelectric force sensors, such as Kistler’s dynamometer, are used to measure the cutting dynamics in terms of the thrust force and the torque. While they are highly accurate and reliable, they are very expensive for small–medium-sized manufacturing shop floors to afford. Also, as the sensor’s dynamic response is affected by workpiece mass and geometry, the sensors measure static forces with potential drifting, causing erroneous force measurements. However, as an inexpensive option, strain-gauge-based mechanical force sensors are becoming a potential candidate, which can still offer reasonably accurate and reliable force measurements. In this case, elastic deformation of a mechanical element is sensed by a series of strain gauges, which are interfaced with an electrical instrumentation, and under loading, the force is measured and estimated as an equivalent electrical voltage output. The gauge sensors are highly sensitive to strain and can be easily attached to the mechanical structure. Recently, the current authors designed and developed an innovative octagonal-elliptical strain gauge-based sensor for measuring milling force data, and demonstrated its working functions [9]. As a simple and robust tool, the designed sensor is found to have a potential to adapt in drilling and evaluate its underlying performance.

With the aid of appropriate sensing and assessment tools, in the past, numerous analytical, numerical, and experimental approaches have been employed to characterize the drilling process, i.e., estimating the thrust force, torque, assessing hole quality, of aluminum alloy and composite materials. In drilling of the fiber-reinforced plastic, Wei et al. [10] reported that the thrust force and hole quality are strongly influenced by the feedrate while the effect of the cutting speed is relatively less. In drilling of Ti6Al4V, Glaa et al. [11] proposed and studied a numerical model in estimating force and torque by taking regenerative chatter and process damping into account, and evaluated the effect of process parameters.

Ko et al. [12,13] studied the effect of drill-bit geometry, suggesting a larger point angle and step drill to enhance the hole quality, i.e., reduced burr size. Similar observations are reported elsewhere in the work by Lauderbaugh [14]. Nauri et al. [15] has investigated tool wear in dry drilling via experimental analysis and optimization, stressing that abrasive and adhesion wear causes tool bluntness and consequently, breakage, thus resulting in the hole’s dimensional inaccuracy. Kurt et al. [16] recommended a low cutting speed and feedrate for enhanced hole quality in the drilling of aluminum alloys.

The effect of the coolant, such as MQL (minimum quantity lubrication) liquid nitrogen, was studied, and it is found that while the coolant reduces the thrust force and tool wear, and improves the hole quality, the use of coolant may cause environmental hazards [17,18]. Along with by a sustainable manufacturing manifesto, the machining process is expected to be less hazardous for the operators, users, and environment. As such, drilling in dry conditions can often be preferred. In an extensive work, Ramulu et al. [19] observed that, in drilling with HSS drill bits, the temperature at the cutting zone increases with the increase of spindle speed and the decrease of feedrate. Increasing the spindle speed leads to increased tool wear, larger entrance and exit burrs, while an increased feedrate leads to an increased thrust force and torque, but smaller entrance and exit burrs. Such observation is somewhat contradictory to earlier findings by Chen and Elhman [20]. It is therefore apparent that the relationships between the drilling parameters and the thrust force, tool wear, and burr formation may vary with the underlying workpiece and drill-bit material. In other words, while the process mechanics, i.e., material removal and chip generation, seems to be generic, the process outcome can still change with the effective parameters and conditions employed [21]. This warrants further investigation to explore and validate such perspective in drilling.

Given the observed discrepancies, with the aid of an in-house designed and built affordable and accurate force dynamometer, the objective of the current study is to recap the drilling mechanics with an aim of comprehensively investigating the effect of the key process parameters—the spindle speed and the feedrate—on the thrust force and torque. As a final outcome, the drilled hole quality, in terms of hole diameter, roundness, surface roughness, and burr formation, is assessed and discussed in relation to the tool wear mechanism and chip formation characteristics. To observe a sustained evolution of tool wear, a series of holes are drilled out of an aluminum 6061 alloy workpiece.

## 2. Materials and Methods

**Figure 1:**
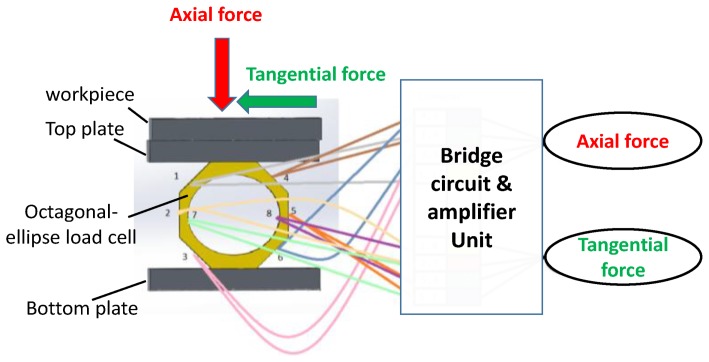
Arrangement of an octagonal-ellipse load cell and the connections of strain gauges to measure axial and tangential forces.

**Figure 2:**
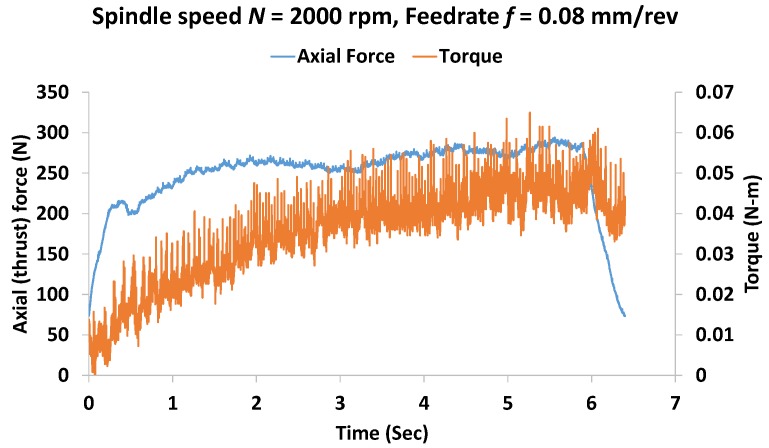
Representative dynamic axial force and torque measurement in drilling of a hole at spindle speed *N* = 2000× rpm and feedrate *f* = 0.08 mm/rev.

Axial force (i.e., thrust force) and torque are two major indicators for the assessment of drilling dynamics. It has been demonstrated that compared to a circular ring, an octagonal structure with an internal elliptical hole generates high strain under loading, thus improving the sensitivity of strain gauge-based load cells [9]. In this study, we have designed and fabricated in-house an octagonal-ellipse shape force dynamometer to measure the thrust force and the torque in drilling. Figure 1 depicts a schematic diagram of the dynamometer structure along with strain gauge arrangement on it. The tangential force data is used to estimate the drilling torque. Top and bottom plates shown are attached to hold the workpiece to be drilled out. The details of the electronics including the bridge circuits and signal processing unit are not shown here for simplicity. The force dynamometer was statically calibrated on an Instron machine (Model: 5567, Norwood MA, USA). A linear relationship between the applied force and the output voltage is found with a fitting accuracy of 98%. To capture the dynamic behavior in drilling, the dynamometer is calibrated in a real drilling test. Figure 2 shows a representative dynamic axial force $\left(F_{a}\right)$ and torque (T) while drilling a hole. Torque is estimated using the relationship of $T=F_{t}*r$ [22], where r = the radius of the drill bit and *F_t_* = tangential force measured by the dynamometer. It is seen that the force and torque vary with time. In particular, torque increases with time as the depth of drilling increases, i.e., when the full contact between the drill bit and the hole surface reaches. This indicates that the force dynamometer used in this study can detect and measuring the dynamic and transient cutting force information.

**Figure 3:**
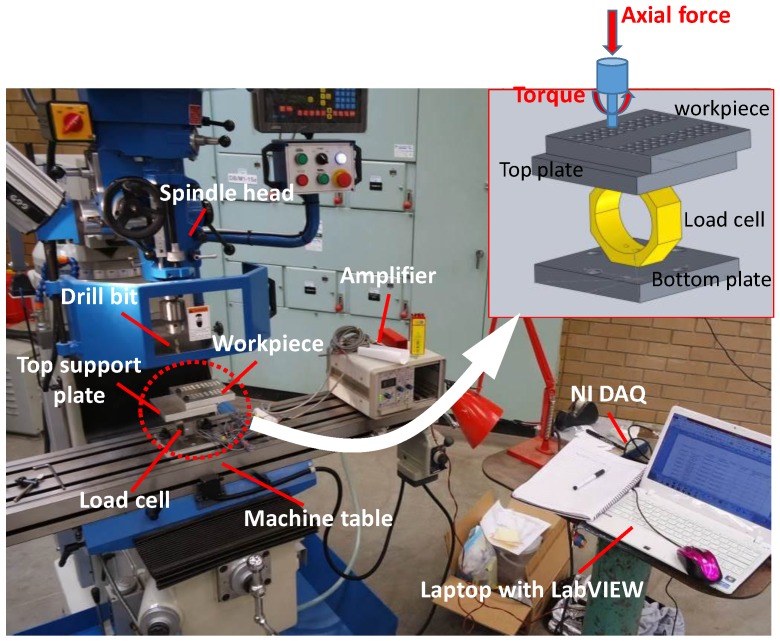
Experimental setup for drilling tests.

To assess hole quality, a series of drilling experiments was conducted on a 3-axis mill-drill machine (MetalMaster’s MB-52VE, HAFCO, New South Wales, Australia). Drilling tests were performed in a dry condition. Parameters considered are shown in Table 1. These levels of parameters are often found to be used in conventional cutting of aluminum alloys in various manufacturing industries [23]. Figure 3 shows an experimental setup including an in-house built force dynamometer along with a data acquisition system. For a given set of process parameters, ten (10) holes are drilled out to investigate the effect of tool wear on hole quality. Therefore, there are six (6) sets of parameter combination made between the spindle speed and the feedrate (see Table 1). For each set of 10 holes, a new and sharp edge drill bit is used. As seen in Figure 3 (see inset image) a dedicated workpiece of 125 mm × 125 mm × 8 mm with pre-drilled holes of 10 mm is made and mounted onto the top plate so that, during drilling of each hole, the axial force is always pointed towards the central axis of the dynamometer. Workpiece is made of aluminum 6061 alloys, whose mechanical properties are shown in Table 2. and the drill bits used are two fluted, TiN-coated A002 high speed steel of 8 mm in diameter along with a cutting angle/drill point = 118°, cutting direction = right-hand. The depth of the hole drilled is 16 mm.

For each hole, the transient forces are measured and recorded using the designed force dynamometer. The transient data with the drilling time are averaged out to determine the final force and torque. Drill-bit cutting edges and chips are observed and analyzed by an optical microscope (Leica’s DVM500) and scanning electron microscope (SEM) (Merlin, Carl Zeiss, Oberkochen, Germany) to investigate tool wear and cutting mechanism as the number of drilled holes increases.

Hole diameter and roundness are measured by a coordinate measuring machine (CMM) (Brown & Sharpe’s MicroXcel 7.6.5) manufactured by Hexagon Metrology (Melbourne, Australia). The machine is connected to measurement software PC-DMIS (Version 3.7), which is used to collect the measurement data for further processing. For each hole, 8 horizontal planes perpendicular to the depth direction from the top of the hole at an interval of 2 mm are chosen, where the CMM probe touches at least 10 points on the inner surface at approximately an equal angle of interval at each depth and measures the diameter on the plane by using the least square circle (LSC) method. CMM Measurement includes entry and exit sides of the hole. Average of the diameter measured at 8 planes is the final diameter. Hole roundness is defined as the radial distance between the minimum circumscribing circle and the maximum inscribing circle, which possesses the profile of the inner surface at a section perpendicular to the axis of rotation. Using the same CMM data obtained for diameter measurement, the final roundness is recorded as the average of roundness measured at 8 depth sections. Drilled surface roughness is measured using a Mitutoyo’s Surftester (Model: SJ 211), where a cut-off length of 2 mm is considered. Roughness (R_a_) measurement is taken on at least five locations along the hole depth direction and their average is recorded as the final value. Burr formation appears to be common in drilling, which affects the hole quality in terms of dimensional accuracy and performance of drilling. In drilling, burrs are generated at the entry and exit side of the hole. In this study, exit burr thickness and height has been measured by the optical microscope (Leica’s DVM 500). Measurements are conducted at four locations equally distant to each other on the hole, and their average is considered the final recorded value.

## 3. Results and Discussion

### 3.1. Axial Force and Torque

**Figure 4:**
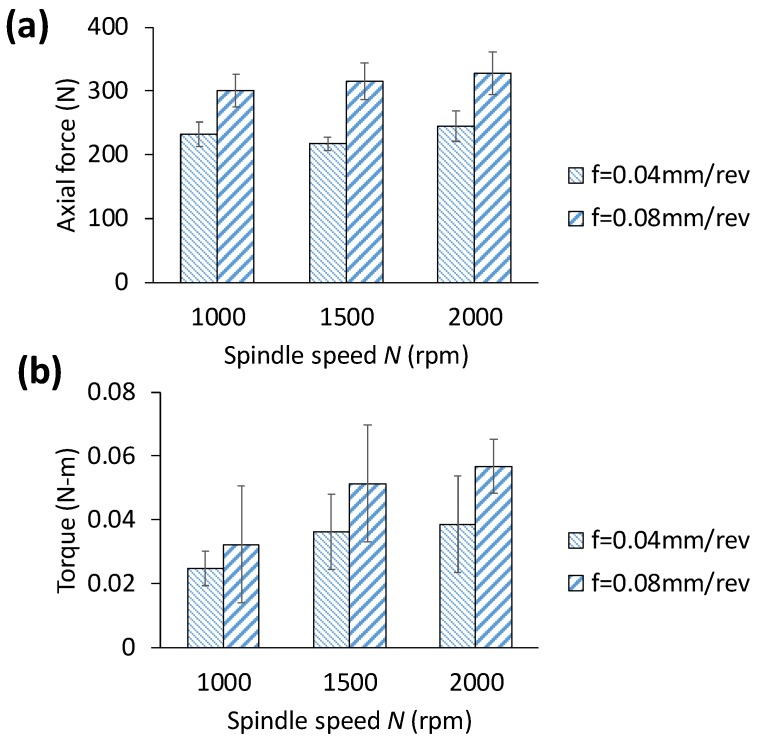
Effect of feedrate and spindle speed on (**a**) average axial force and (**b**) average torque. Error bar indicates standard deviation of force data for drilling of 10 holes.

Figure 4 shows the variation of average axial (thrust) force and torque with respect to spindle speed and feedrate. For each hole, the average value estimated as the average of transient force data measured from the moment when the drill bit enters full into workpiece until the bit exits the hole completely. Clearly, both axial force and torque increase significantly with the increase of feedrate. For instance, when feedrate is increased from 0.04 mm/rev to 0.08 mm/rev, the increase of axial force is 29.23%, 44.94% and 34.02% at spindle speed of 1000× rpm, 1500× rpm and 2000× rpm, respectively. For the same change of feedrate, torque increases by 29.95%, 41.55% and 47.65%, at spindle speed of 1000× rpm, 1500× rpm and 2000× rpm, respectively. Larger feedrate means the drill bit experiences faster penetration axially, thus resulting in larger chip thickness and material removal rate. As a result, thrust force and torque increase. On the other hand, at a given feedrate, spindle speed appears to have insignificant influence on thrust force. For example, at a feedrate of 0.08 mm/rev, axial force increases by 4.74% and 9.18% when the spindle speed changes from 1000× rpm to 1500× rpm, and to 2000× rpm, respectively. In drilling of homogenous titanium alloy stacks, Wei et al. [10] observed that the change of thrust force with respect to spindle speed is minimum or negligible. Hence, this supports our results for drilling of aluminum alloys. In other words, drilling of titanium and aluminum alloys follow qualitatively the similar trend, and so is expected in terms of hole quality.

However, some moderate effect on torque is noticed. For instance, at a feedrate of 0.08 mm/rev, torque increases by 59.19% when spindle speed increases from 1000× rpm to 1500× rpm, and then remains nearly stable with a moderate increase of 10.95% as the spindle speed reaches to 2000× rpm. As the spindle speed increases further, temperature generated at the cutting zone softens the material, and hence, the drill bit requires less force for plastic deformation and shearing of material. As can be seen in Figure 4a, a slight increase of axial force (and torque) with increase of spindle speed can be due to the variation of degree of thermal softening and temperature rise because of actual spindle speed variation (i.e., commanded spindle speed may not be constant during drilling). The results clearly suggest that higher spindle speed and lower feedrate may be preferred; but tool wear effect, which will be discussed in the following section, must be taken into consideration simultaneously.

### 3.2. Tool Wear Mechanism

**Figure 5:**
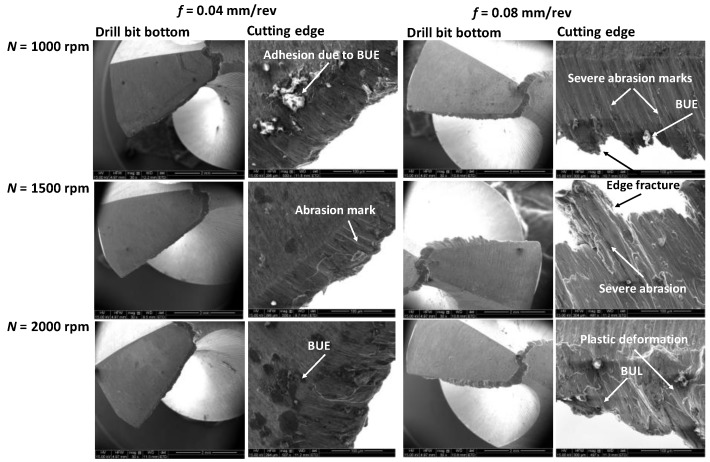
Scanning electron microscope (SEM) images of the drill-bit bottom and their magnified view of the cutting edge after 10th hole for different spindle speed and feedrate.

Figure 5 illustrates SEM photos of the drill bit’s cutting edges after 10th hole for each combination of spindle speed and feedrate. As compared to the chisel edge, the cutting-edge wear is the dominant factor impacting the drilling performance. Noticeable wear on the flank face includes adhesion due to built-up edge, abrasive and chipping or fracture. These types of wear are very common for cutting of soft material, such as, aluminum alloys. It can be seen from Figure 5 that, adhesion wear has been the obvious wear mechanism, regardless of the spindle speed and the feedrate studied. As the feedrate increases from 0.04 to 0.08 mm/rev, the abrasion wear takes place, causing the true flank wear and weakens the strength of the cutting edge. Large feedrate means higher material removal rate, causing larger thrust force onto the cutting edge. Consequently, the edge chipping along with plastic deformation starts to occur, which may lead to the breakage of the drill bit. In particular, TiN coating on the drill bit would be more vulnerable. This observation is consistent with the findings of drilling force and torque. It can, therefore, be imperative to say that the moderate feedrate can be recommended to avoid early initiation of the cutting-edge wear and failure. Though no significant measurable wear on the flank face is observed even after the 10th hole, it is expected that the severity of the cutting-edge wear will accelerate as the drilling time for producing more holes will increase. In addition to shear straining in primary shear zone, the complex interaction and temperature rise at the interface between the tool and chip rule the dominant adhesion wear evolution in drilling. Nuoari et al. [15] reported adhesion occurs in two stages as built-up edge (BUE) and build-up layer (BUL) as the drilling of more holes continues. Initial unstable BUE transforms into BUL due to pressure and temperature in contact zone, leading to potential diffusion of aluminum towards the tool, and micro-welding forms on tool surface. When BUL formation reaches tool edge and breaks due to dynamic non-continuous cutting, the tool edge becomes irregular and weakens, which potential may result in catastrophic fracture failure. SEM images shown in Figure 5 show clearly a change of BUE to BUL along with rough tool edges. As such, our results on tool wear are consistent with literature. Therefore, along with appropriate choice of spindle speed and feedrate, the use of highly wear resistant and low friction coated drill bit (e.g., (Ti-Al)N or diamond coating via CVD/PVD on tungsten carbide (WC) tool [24]) along with an effective cooling mechanism can be considered to minimize the severity of tool wear, and hence, improve drilling performance in terms of hole quality (which is discussed in the following sections), tool life and manufacturing productivity [15].

### 3.3. Hole Diameter

**Figure 6:**
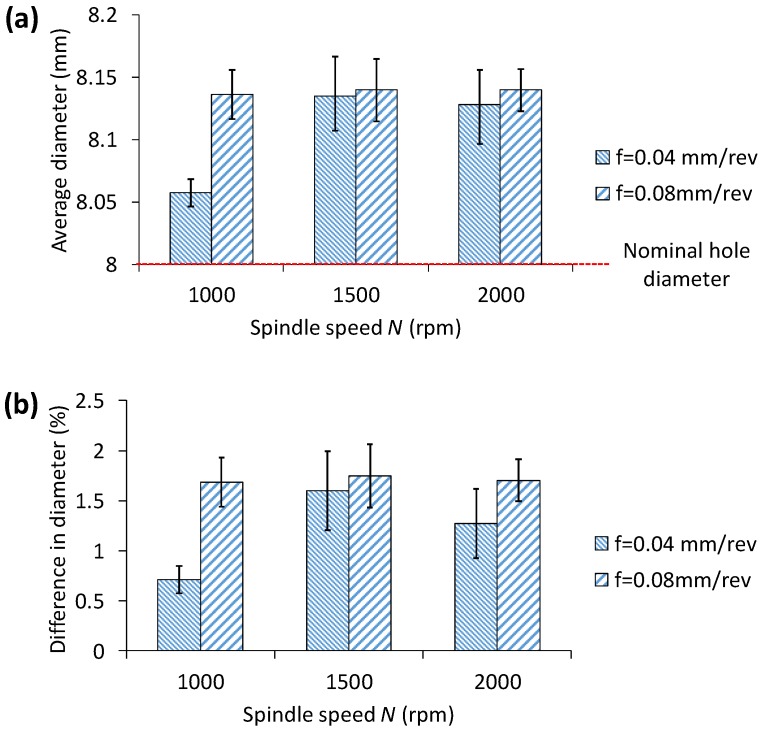
Effect of federate and spindle speed on (**a**) hole diameter and (**b**) % of difference with respect to its nominal size (=8 mm). Error bar indicates standard deviation of hole diameter for drilling of 10 holes.

Figure 6 shows the variation of average hole diameter and % of difference from its nominal size with respect to spindle speed (*N*) and feedrate (*f*). As is obvious, for the range of speed and feedrate studied, hole size is always larger than the nominal and the maximum % of difference in diameter is less than 2%, i.e., the hole is less than 150 µm large from its nominal dimension (of 8 mm in diameter). Despite the diameter increase is relatively small, it appears that smaller feedrate is shown to reduce the dimensional difference while the spindle speed has no noticeable effect, except for the condition of *N* = 1000× rpm and *f* = 0.04 mm/rev, which indicates that low speed and low feedrate would be preferred. Lower feedrate means slower penetration rate and the cutting edge removes material with smaller chip thickness, allowing a stable and jerk-free drilling, and as a result, the hole diameter with less dimensional error is achieved. It is reported that faster spindle speed causes temperature rise at the cutting zone, and softens the material, thus facilitating a smoother drill surface with a good surface quality with an improved dimensional accuracy. Though the difference is not statistically significant, our results on hole diameter shown in Figure 6, indicate an improvement of dimensional accuracy as the spindle speed increases from 1000× rpm to 2000× rpm. The results are consistent to force and torque data. At a low feedrate, shear cutting is the dominant mechanism, resulting in a continuous chip generation and lower force, and as a result, hole deviation is minimum. Similar conclusions on hole size in drilling of Al alloys are observed elsewhere in literature [2,23]. Therefore, it is safe to say that, given a spindle speed, slower feedrate can suitably be selected to minimize the dimensional error. Although a slower feedrate compromises productivity, the decision must be made by establishing a fair balance between the productivity and the dimensional accuracy required.

### 3.4. Burr Formation

**Figure 7:**
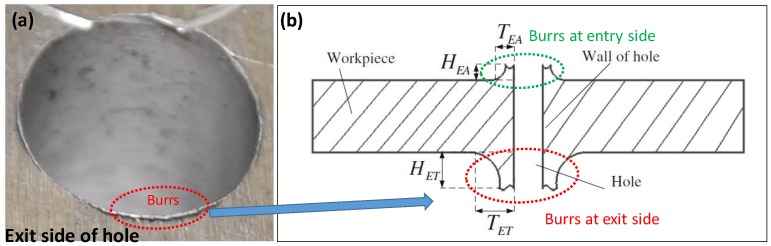
(**a**) Representative hole with burrs at the exit side of a hole (**b**) definition of burr geometry.

**Figure 8:**
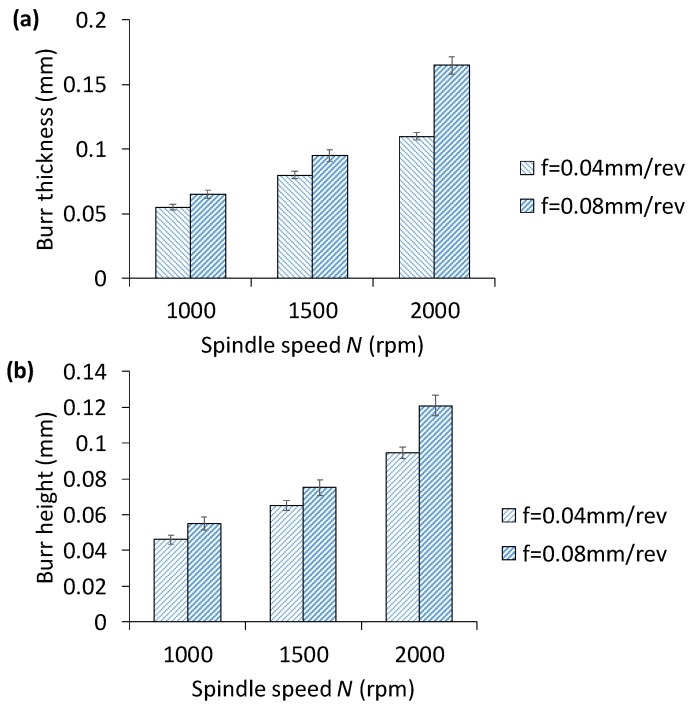
Effect of spindle speed and feedrate on (**a**) thickness and (**b**) height of exit burr. Error bar indicates standard deviation of burr size for drilling of 10 holes

**Figure 9:**
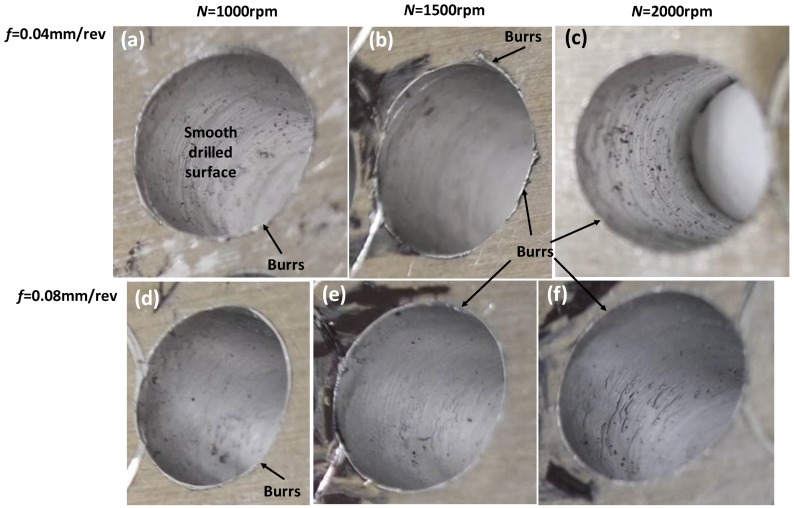
Representative topography of exit side of holes at parameter combination of (**a**) *N* = 1000 rpm and *f* = 0.04 mm/rev, (**b**) *N* = 1500 rpm and *f* = 0.04 mm/rev, (**c**) *N* = 2000 rpm, *f* = 0.04 mm/rev, (**d**) *N* = 1000 rpm, *f* = 0.08 mm/rev, (**e**) *N* = 1500 rpm, *f* = 0.08 mm/rev (**d**) *N* = 2000 rpm, *f* = 0.08 mm/rev.

Figure 7 shows an example drilled hole with burrs at the exit side, and the geometric definition. Burr size at the entry side is found to be smaller than the exit side. Therefore, burr at the exit side is measured for drilling of ten holes at each combination of process parameters and presented for analysis. Figure 8 shows the change of the exit burr thickness and height with respect to spindle speed and feedrate. It can be seen that higher spindle speed and feedrate increase the burr size. In particular, the increase of burr size with the feedrate is higher when the spindle speed is larger. For instance, the change of burr thickness between feedrate of 0.04 mm/rev and 0.08 mm/rev increases from 18% to 50% when the spindle speed increases from 1000× rpm to 2000× rpm, respectively. On the other hand, for the same condition, the burr height jumps from 19.56% to 28%. As explained in earlier section, higher feedrate rate introduces higher thrust force, which causes larger and faster chip generation, and, as a result, the burr geometry becomes larger. Overall, spindle speed influences the burr thickness the most than the burr height. Figure 9 shows a representative topography of the exit side burr with respect to spindle speed and feedrate. It is to be noted that burr formation and its increase can be carefully observed when drilling with larger diameter. In other words, larger diameter tool increases cutting speed and dynamic rake angle, which cause plastic deformation in machining hardening layer and residual stress depth on the hole wall, hence resulting in increase of burr thickness and height [25]. The above suggests that lower spindle speed and feedrate must be chosen to minimize burr generation, thus saving cost for further rework in removing burrs. Past computational modelling and experimental investigation on drilling of aerospace aluminum and composite materials have reiterated the severity of burr formation, and made similar recommendations to ensure superior hole quality [26]. For instance, Sorrentino et al. [22] reported a reduction of push-out delamination factor (i.e., exit burr geometry) by 37% for drilling of CFRP (carbon fiber-reinforced polymer) when feedrate is changed from 0.3 mm/rev to 0.1 mm/rev.

### 3.5. Roundness and Roughness

**Figure 10:**
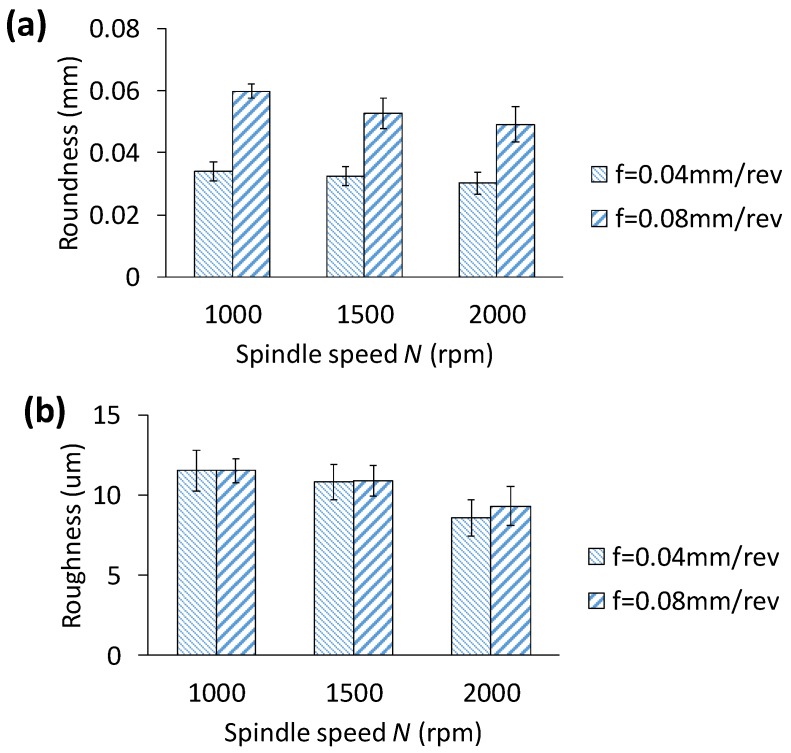
Effect of feedrate and spindle speed feedrate on (**a**) hole roundness and (**b**) surface roughness.

Figure 10a show the roundness error with respect to spindle speed and feedrate. The roundness error increases significantly with the feedrate. For instance, when the feedrate is increased from 0.04 mm/rev to 0.08 mm/rev, the roundness increases by 78.78% at a spindle speed of 1000× rpm. The roundness error could primarily be because of burrs generated at the entry and the exit sides of the hole, the abrupt thrust force, and the dynamic instability of the drill bit. Higher thrust force due to a larger feedrate would be the dominant reason for an increased roundness error. For all ten holes drilled out, the roundness error is less than 60 µm, which is reasonably acceptable for small to medium size holes (of 8 mm diameter). On the other hand, the roundness error is less impacted by the spindle speed, but reduces at a large feedrate of 0.08 mm/rev. This is surprisingly interesting observation though, and conflicts with the trend of burr geometry with higher feedrate and spindle speed. Such variation could be due to the errors in roundness measurements by CMM. In other words, as the CMM probe touches the inner surface of the hole, the cutting debris potentially adhered to the surface affects the measurement, and hence, the overall roundness error. Even though hole’s inner surface is cleaned by high speed air spray by an air gun, very minute debris may be stick to the surface. Effect of spindle speed and feedrate on hole roughness R_a_ is shown in Figure 10b. It is seen that roughness varies between 8.5 µm and 11.15 µm. Feedrate has no or minimum influence on roughness, while higher spindle speed is shown to give lower roughness. These results imply that low to moderate spindle speed and feedrate can safely be selected to achieve smooth hole surface finish. Clean and smooth hole surface is expected to pull-out strength and mechanical integrity of the underlying assembly structure. Observation of chip morphology which is shown in the next section further explains the mechanism for improved surface finish.

### 3.6. Chips Formation

**Figure 11:**
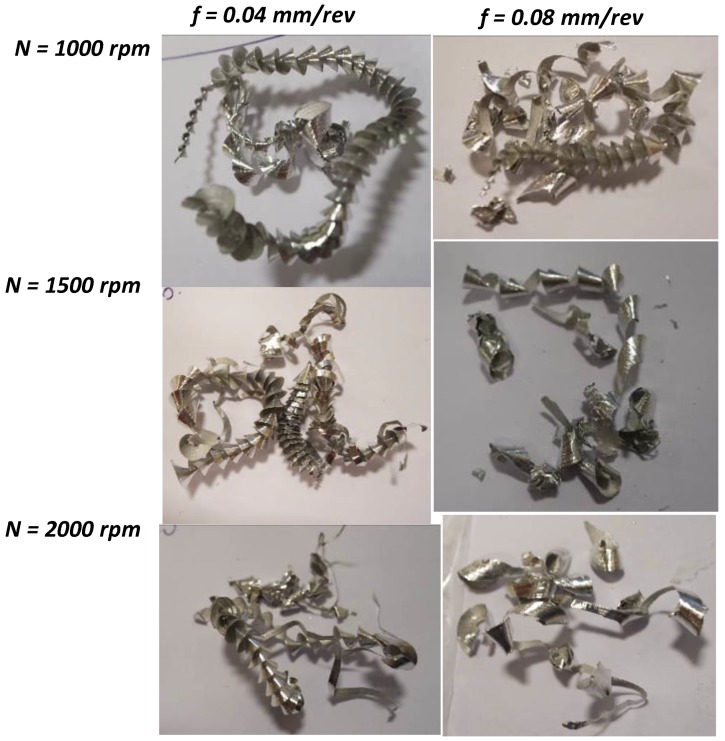
Cutting chips after drilling of 10th hole at different spindle speed and feedrate.

Figure 11 shows cutting chips after 10th hole at different combination of spindle speed and feedrate. In most cases, the chips are continuous, entangled curly shape. It appears that the spindle speed has less influence on the chip generation, while the feedrate affects the most. As the feedrate increases from 0.04 to 0.08 mm/rev, the chips are not always continuous, but fractured and broken. Reduced edge sharpness due to wear at high feedrate is responsible for the broken and segmented chips. In other words, because of wear, the interaction between the rake face and the workpiece changes, which may result in the segmented chip generation. Also, when feedrate increases, shearing section becomes larger and chips become wider. Therefore, chips struggle to wind continuously due to large stiffness, and hence start to break into small segmented and/or spiral pieces. Furthermore, high spindle speed means high kinetic energy into chips, which may cause chip breakage at higher feedrate (Figure 11). In other words, chips flow through the flutes experience tremendous resistance due to the contact friction and break away. The similar finding is observed and reported via experimental and computational studies on machining of aerospace aluminum alloys [10,27]. It is to be noted that while segmented chips are favorable for easy evacuation and management of chips, the underlying process often deteriorate the generated hole quality. Therefore, the selection of drilling process parameters must be considered according to the desired hole-quality requirements, e.g., hole dimension, roundness, and finish.

## 4. Conclusions

This paper presents an experimental investigation on the evaluation of hole quality in drilling of aluminum alloys. Compared to spindle speed, feedrate is the most dominant parameter, significantly affecting drilling behavior. For given spindle speed range, maximum increase of axial force and torque is 44.94% and 47.65%, respectively when feedrate increases from 0.04 mm/rev to 0.08 mm/rev. Stable, jerk-free cutting at feedrate of as low as 0.04 mm/rev is shown to result in hole dimensional error of less than 2%. Results of burr geometry and roundness follow the same trend, while roughness is minimally influenced by both spindle speed and feedrate. Built-up edge followed by abrasion and micro-chipping at the cutting edge produce noticeable wear mechanism, and their consequence may accelerate as the number of drilled holes further increases. This result is supported by chip morphology observation, i.e., more broken and segmented chips are noticed at a higher feedrate, as opposed to the continuous entangled chips at a lower feedrate.

It should be noted that coolant [27] and change of tool geometry [28], which may affect the drilling performance, is not taken into account in this study. While both may quantitatively change force and hole-quality metrics presented, it is expected that the qualitative trend will remain the same, and, as such, so do the conclusions of the paper.

## Figures and Tables

**Table 1:** Drilling parameters.

Parameters	Values
Spindle speed *N* (rpm)	1000, 1500, 2000
Feedrate *f* (mm/rev)	0.04, 0.08

**Table 2:** Mechanical properties of workpiece material (Al 6061 alloys) used.

Parameters	Values
Young’s modulus (GPa)	68.9
Poisson’s ratio	0.33
Tensile strength (MPa)	124–290
Density (g/cm^3^)	2.7
Thermal conductivity (W/m.K)	151–202
Specific heat capacity (J/Kg·K)	897
